# Supplementary material for: Replication of Human Sapovirus in Human-Induced Pluripotent Stem Cell-Derived Intestinal Epithelial Cells
Source: Viruses. 2023 Sep 15;15(9):1929. doi: 10.3390/v15091929 (PMC10536750; doi:10.3390/v15091929)
Supplement: Supplementary file 1 [file viruses-15-01929-s001.zip › viruses-2563125-supplementary.pdf]

Supplementary Figure S1

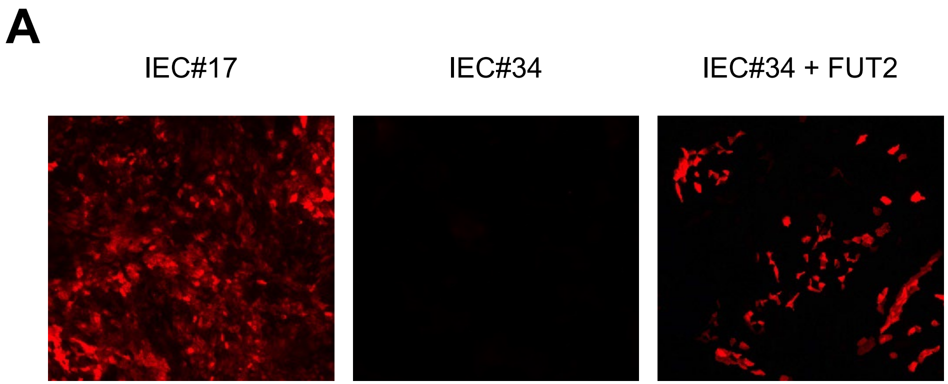

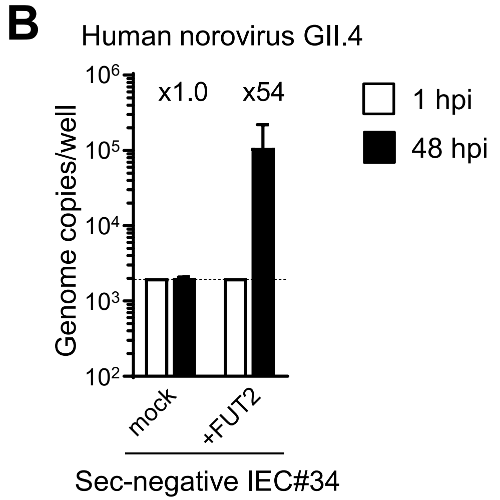

Supplementary Figure S2

A

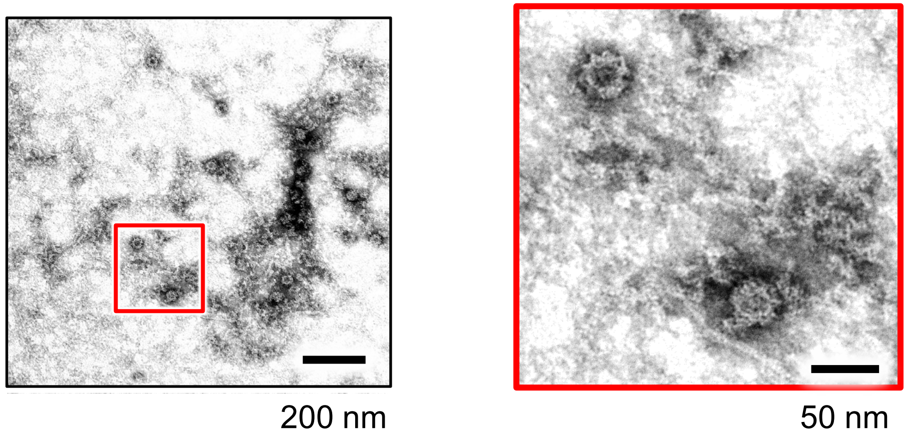

B

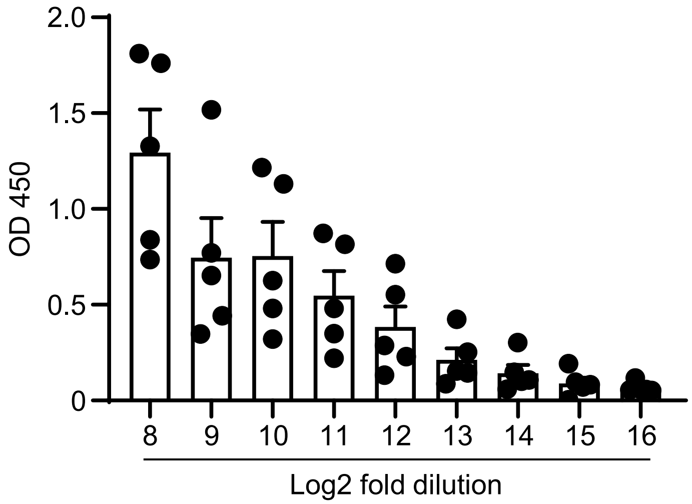

Supplementary Figure S1. Requirement for α(1,2) fucose during the in vitro replication of human norovirus GII.4. (A) Monolayers of human iPSC-derived IECs were cultured for 6 days and were then stained with *Ulex europaeus* agglutinin-1 (UEA-1; red). Scale bars, 100 μm. (B) Monolayers of secretory-negative IEC#34 or *FUT2* gene-introduced IEC#34 were inoculated with 2×10^6 genome equivalents of the GII.4 genotype of human norovirus (Sample ID 17B93 [11]). Inoculation and sampling were performed as described in the Methods section. Viral genome RNA was extracted from supernatants sampled at 1 or 48 hpi, and genome equivalents were then quantified with RT-qPCR. Each value is representative of three independent experiments and is shown as the mean ± SD from four wells of supernatants for each culture group. The mean fold changes in viral genome between the two time points are indicated above each black bar (48 hpi).

Supplementary Figure S2. Preparation and of GII.1 HuSaV VLP and antiserum

against the VLP. (A) Transmission electron microscopy analysis of GII.1 HuSaV VLP. Purified VLP was negatively stained with 1 % uranyl acetate solution. Scale bars indicate 200 nm (left panel) and 50 nm (right panel). (B) Homotypic titers of anti-GII.1 HuSaV antiserum diluted at the indicated magnification were evaluated by ELISA. Data are means ± SD (*n*= 5).
